# A 4-Year Retrospective Analysis of Salivary Gland Cytopathology Using the Milan System for Reporting Salivary Gland Cytology and Ancillary Studies

**DOI:** 10.3390/cancers11121912

**Published:** 2019-12-01

**Authors:** Charlotte Dubucs, Céline Basset, Dominique D’Aure, Monique Courtade-Saïdi, Solène M. Evrard

**Affiliations:** 1 Department of Cytology and Pathology, Toulouse Cancer University Institute, CHU Toulouse, CEDEX, 31059 Toulouse, France; Dubucs.Charlotte@iuct-oncopole.fr (C.D.); basset-leobon.c@chu-toulouse.fr (C.B.); daure.d@chu-toulouse.fr (D.D.); monique.courtade-saidi@univ-tlse3.fr (M.C.-S.); 2 Rangueil Faculty of Medicine, Paul Sabatier University, Toulouse-3, CEDEX, 31062 Toulouse, France; 3 INSERM UMR 1037, Centre for Cancer Research of Toulouse, 31062 Toulouse, France

**Keywords:** salivary gland cytopathology, fine needle aspiration, The Milan System for Reporting Salivary Gland Cytopathology, immunocytochemistry, FISH

## Abstract

The cytopathology of salivary glands presents major challenges due to the heterogeneity of benign and malignant neoplasms, which is reflected in the large range of WHO 2017 Classifications. Fine needle aspiration (FNA) of salivary gland tumours is still the favoured initial approach as it results in good sensitivity and specificity. The Milan System for Reporting Salivary Gland Cytopathology (MSRSGC) was published in 2018 and comprises seven categories. We report results from a 4-year retrospective analysis of 328 salivary gland FNAs which were reviewed and classified according to the MSRSGC. We assess the risk of neoplasm, the risk of malignancy and the contribution of ancillary studies to the diagnosis. Benign neoplasms were the most frequent diagnosis (44.2%). Malignant and suspicious for malignancy were identified in 11.3% and 4.9% of diagnosed cases, respectively. Histopathological analysis after surgery was available for 216 (65.8%) of the cases. All malignant cases were confirmed post-surgery, and 68.8% of suspicious for malignancy were confirmed as malignant tumours. Immunocytochemistry was informative in 72.3% of cases. Immunocytochemistry and FISH provided the definitive diagnosis in 23.7% and 33% of cases, respectively. In conclusion, the MSRSGC is more effective when specific features of neoplasms can be identified. Ancillary studies help to further characterise salivary gland tumours and thereby increase the accuracy of MSRSGC.

## 1. Introduction

The cytopathology of salivary glands presents major challenges due to the heterogeneity of benign and malignant neoplasms, which is reflected in the large range of World Health Organisation 2017 Classifications [1]. Fine needle aspiration (FNA) of salivary gland tumours is still the favoured approach as it results in good sensitivity (83–92%) and specificity (93–100%) [2]. Although the most commonly occurring neoplasms are relatively easy to identify from cytological specimens, some tumours are heterogeneous and/or share overlapping features, and are challenging to assign to specific categories. FNA is predominantly used to determine before surgery whether the lesion is neoplastic or not, whether the neoplasm is benign or malignant, and to differentiate between an epithelial neoplasm and a lymphoma or between a primary neoplasm and a metastasis. The Milan System for Reporting Salivary Gland Cytopathology (MSRSGC) was published in 2018 and was drawn up by the American Society of Cytology and the International Academy of Cytology [3]. It standardises terminology and stratified salivary gland lesions into seven categories: non-diagnostic (ND), non-neoplastic (NN), atypia of undetermined significance (AUS), benign neoplasm (BN), salivary gland neoplasm of uncertain malignant potential (SUMP), suspicious for malignancy (SM) and malignant (M). Even if the MSRSGC classification does not require a precise identification of the neoplasm, however it should be indicated whenever feasible mostly for BN and M categories. In M category, it is recommended to specify if the malignancy is either low-grade (LG-M) or high-grade (HG-M) as the malignancy grade is clinically relevant in order to plan extension of the surgical treatment. The MSRSGC has shown an accuracy of 0.67 for HG-M [4] which can be improved when associated with clinically or pathologically substantiated cervical metastases (0.77). Moreover, the precise cytological identification of a neoplasm helps to determine whether it is benign or LG-M. This is not always possible based on morphology alone and in some cases, ancillary studies may be required to establish a definitive cytological diagnosis. The latter are described in the MSRSGC and predominantly include immunocytochemistry (ICC) and fluorescence in situ hybridisation (FISH). Indeed, some tumours contain specific translocations, which can be easily identified from cytological specimens [5]. We report findings from a 4-year retrospective analysis of salivary gland FNA, which were reviewed and classified according to the MSRSGC. We also assess the risk of neoplasm (RON) and of malignancy (ROM) and the contribution of ancillary studies to the definitive diagnosis.

## 2. Results

### 2.1. Classification of Salivary Gland Cytology Using the Milan System for Reporting Salivary Gland Cytopathology

Between February 2014 and March 2018, 328 salivary gland FNAs from 314 patients were analysed in our laboratory. Fourteen cases originated from accessory salivary glands (4%), 291 (89%) from the parotid and 23 (7%) from the sub-mandibular glands. The median age of patients was 64 years (range 11–93) and the male/female ratio 1.17.

We received the majority of FNAs as air-dried smears with a median of 4 slides and a mean of 3.71 ± 1.87 slides per case. In 37 cases (11.28%) a liquid based cytology (LBC) was also associated. Only 3 cases were addressed in LBC alone.

**Figure 1:**
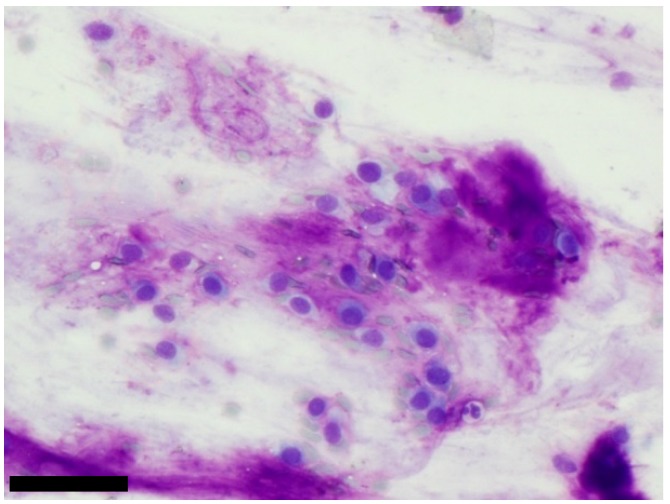
Benign neoplasm: a typical pleomorphic adenoma with plasmacytoid cells mixed with a fibrillary matrix which stains magenta on MGG, (scale bar: 100 µm).

Results were reviewed and classified using the MSRSGC (Table 1). The most frequent diagnosis was BN (44.2%) with a majority of pleomorphic adenoma (PA) diagnoses (27,1%) (Figure 1). Moreover, 55 (16,8%) Warthin’s tumours and 1 (0,3%) schwanomma were also included in the BN category. The ND category represented 25.6% of cases. We were unable to formulate a diagnosis in 84 cases (ND category) mainly due to cystic content or because of pauci-cellularity of the sample (25 (7.6%) and 55 (16.8%) cases respectively). The four last cases only contained a few lymphocytes without any epithelial cells and were thus classified as ND. About 8% of cases were reported to be NN. Most of these cases were reactive lymph nodes with inflammatory cells (12/27 cases, 44%). Normal lymph nodes (seven cases), cysts (seven cases) and sialadenitis with lithiasis (1 case) were the three other cytological diagnoses described in NN category. Malignant lesions (primary as well as secondary) represented 11.3% of cases, among these 2.7% were LG-M, 62.2% were HG-M and 35.1% were solid tumour metastases. Almost 5% of cases were reported to be SM. They were predominantly cases of suspected lymphomas (10 cases; 62.5%). SUMP represented almost 5% of the cases. As described previously, SUMP with oncocytic cells or without any specific morphological features were separated into oncocytic subtype (7/15) or unspecified subtype (8/15), respectively [6]. No SUMP case with basaloid cells or basaloid features was described in our study. Finally, only 1.2% of the cases were reported in AUS category.

### 2.2. Correlation of Cytological Results with Histology

**Figure 2:**
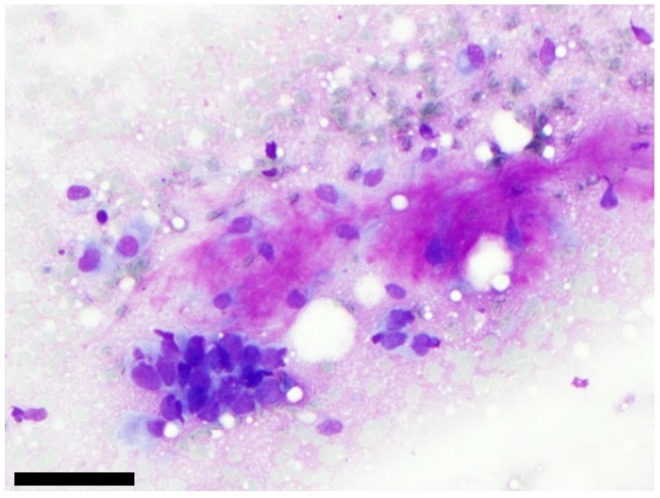
Example of one case classified as SUMP with unspecified features after cytological analysis and diagnosed as poorly differentiated carcinoma after surgery. On this picture, we can see a cluster of atypical cells with high nuclear/cytoplasmic ratio which is close to a fibrillary matrix (MGG staining). FISH with *PLAG1* or *MYB* probes were negative, (scale bar: 100 µm).

Post-surgery histopathological analyses were available for 216 cases (65.9%) (Table 1). The majority of BN were confirmed after surgery (100% of Warthin’s tumours and 94.4% of PA). Among cases classified as NN on cytopathological analysis, only one was categorised as a benign neoplasm (Warthin’s tumour) after histological examination. Among the four cases classified as AUS, only two of these had surgery with histopathological analyses. Both of these cases were definitively diagnosed as benign neoplasms (one PA and one cystadenoma). Regarding the SUMP category, 7/15 corresponded to cytological cases with oncytic features among which two were finally diagnosed as oncocytic hyperplasia, one as oncocytic PA, and one as a high-grade salivary duct carcinoma. The eight remaining cases corresponded to cytological cases with unspecificied features among which four were malignant (one polymorphous low-grade adenocarcinoma, one secretory carcinomas, one adenoid cystic carcinoma and one poorly differentiated tumour (Figure 2) and four cases were non-malignant (one Warthin’s tumour, one fibrous nodule, one benign cyst and one atrophic sialadenitis). The SM category was separated into suspicious for lymphoma (10/16) or suspicious for solid tumour (6/16). The diagnostic of lymphoma was confirmed in 8/10 cases. One case corresponded to a reactive lymphocytic infiltration of the parotid gland and the last case corresponded to a lymphoepithelial cyst in a HIV+ patient (we were blind of the clinical data for the cytological analysis). Among SM cases suspicious for solid tumours, 3/6 corresponded to malignant tumours (one mucoepidermoid carcinoma, one myoepithelial carcinoma and one salivary duct adenocarcinoma) and 3/6 corresponded either to PA (2/3) or a myofibroblastic granuloma. All malignant neoplasms were confirmed after surgery. The RON and the ROM for each category are indicated in Table 1.

### 2.3. Ancillary Studies Help to Further Characterise Salivary Gland Tumours

**Figure 3:**
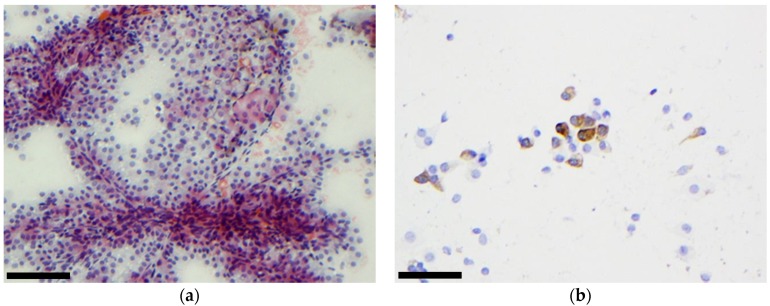
Malignant tumour: Secretory carcinoma. (**a**) On Papanicolaou staining we observed a high cellular content, papillary structures and the presence of cells with foamy cytoplasm, (scale bar: 500 µm); (**b**) ICC performed on smears revealed that cells were positive for mammaglobin, (scale bar: 100 µm).

**Figure 4:**
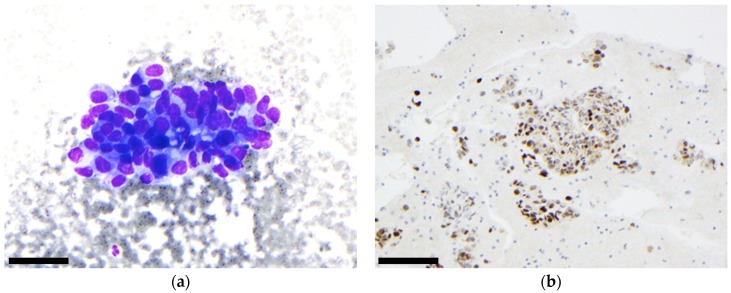
Malignant tumour: metastasis from lung adenocarcinoma (**a**) The cells had high N/C ratio but did not show any specific differentiation (MGG staining; scale bar: 100 µm); (**b**) ICC performed on a cellblock revealed that cells were positive for TTF1 (scale bar: 200 µm).

ICC or FISH was performed in 76 and 48 cases, respectively. Results are shown in Table 2. ICC was performed predominantly on smears (74/76 cases). Only two cases provided enough material to perform a cellblock used for ICC. The ICC did not contribute to the diagnosis in 27.6% of cases, due to lack of material, technical problems or due to the incorrect choice of antibody to confirm the definitive diagnosis (particularly when not enough slides were available for the analysis). In nine cases (11.8%), the ICC helped to eliminate the diagnosis of a metastasis. This is particularly useful in cases previously diagnosed with a primary tumour. In fact, as shown in Table 3, among these nine cases, a medical history of neoplasm was known in three cases. The choice of antibodies used was thus defined accurately. For the six other cases, we used antibodies to eliminate a metastasis of a carcinoma (anti-AE1/AE3 antibody) or of thyroid or lung carcinoma (anti-TTF1 antibody). In 36.8% of cases, ICC helped to differentiate a BN or LG-M lesion from a HG-M lesion, predominantly with the anti-Ki67 antibody. In 18 cases (23.7%), ICC confirmed the diagnosis (Figure 3 and Figure 4). Among the 13 malignant lesions diagnosed as metastasis of solid tumours, information about a primary tumour was known in 10 cases and ICC was performed in 5/10 cases. In 4/5 cases ICC provided the diagnostic. In the last case, ICC did not contribute to diagnosis due to lack of tumour cells on the slide.

**Figure 5:**
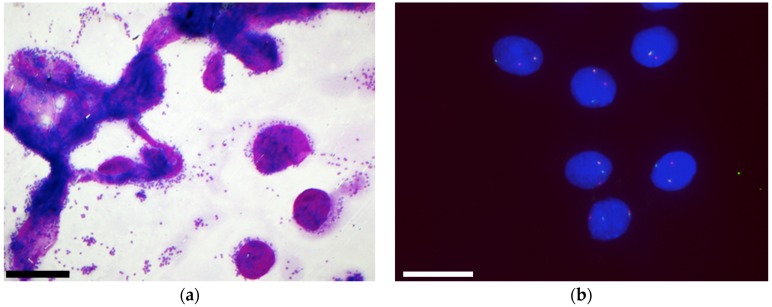
Malignant tumour: adenoid cystic carcinoma: (**a**) On the MGG staining, we observed the typical tubular and hyaline globules, (scale bar: 500 µm); (**b**) FISH performed on the same case revealed rearrangement of the *MYB* gene as indicated by a split between the green and red signal, (scale bar: 40 µm).

FISH was always performed on smears as previously described [5]. It did not contribute to the definitive diagnosis in 21% of cases (due to the interference of background staining or insufficient material). FISH was uninformative in 46% of cases, due to negative results. It provided the definitive cytological diagnosis in 33% of cases. FISH was particularly helpful for diagnosing malignant tumours (mucoepidermoid carcinoma with a *MECT1-MAML2* fusion, adenoid cystic carcinoma with a *MYB* translocation and secretory carcinoma with an *ETV6* translocation) (Figure 5).

In 11 cases, we received enough slides to perform both ICC and FISH (Table 4). Six of them were BN cases (all PA), one was a SUMP case and four were M cases (one low-grade and three high-grade). When FISH is negative, ICC may help at least to differentiate BN/LG-M from HG-M using anti-Ki67 antibody and sometimes may provide the diagnosis, especially in metastasis.

## 3. Discussion

In this study, we retrospectively classified all cases of salivary gland fine needle aspirations analysed in our laboratory between 2014 and March 2018, according to the MSRSGC. A high proportion (25.6%) of cases were considered ND. This is somewhat higher than that reported in other studies by Park et al., (9.9%), Rohilla et al., (2.2%), Savant et al., (9.2%), Song et al., (13.5%), Viswanathan et al., (12%) and Wei et al., (2%) [4,7,8,9,10,11]. This may be related to the method of sample collection. Indeed, most of the samples from our institution were collected at the initial patient consultation, during which the clinician performed the FNA without ultrasound-guidance. In fact, we previously showed, as did others, that ultrasound guided FNA increases the quality of the sample collected when compared to FNA performed in the absence of ultrasound guidance [12,13]. This may explain the high frequency of samples with cystic content and without any informative epithelial cells observed, and also why the ROM in the ND category of our study is higher than in the MSRSGC (34% vs. 25%) [3]. We also note that 4/84 samples from ND cases only contained few lymphocytes and turned out to be lymphomas. In these cases, the number of lymphocytes was too low or the cells were insufficiently preserved to provide the diagnosis.

The NN category represented 8.2% of cases. Most non-neoplastic lesions are clinically evident and the FNA is not systematically performed in these cases. In our study, 9 cases included in the NN category were analysed by histopathology with none of these cases finally diagnosed as malignant. The AUS category only comprised four samples, with only two of these undergoing surgery and therefore histopathological analysis. Both cases were finally diagnosed as benign neoplasms (one PA and one cystadenoma) and the ROM cannot be interpreted for this category. The majority of our cases were classified as BN. Indeed, PA and Warthin’s tumours were the most frequent diagnoses (respectively 27.1% and 16.8% of total cases). This is consistent with the frequency reported in the literature [9,10,14,15]. These diagnoses are indeed straightforward for representative samples. However, four cases of PA identified on cytological samples turned out to be malignant after pathological examination. Among these four cases, three of them turned out to be carcinomas ex PA. These lesions can be difficult to detect on cytological samples due to the absence of the carcinoma component in the FNA. The last false negative PA case turned out to be an adenoid cystic carcinoma. In fact, adenoid cystic carcinoma is one of the differential diagnosis when highly cellular FNA sample is observed with scant matrix, which was the case here [3]. Regarding the SUMP category, it represents only 4.6% of the cases and half of them was composed of cells with oncocytic features. Two of the oncocytic SUMP cases were finally diagnosed as neoplasm (1/4 benign and 1/4 malignant) after pathological examination. The two other oncoyctic SUMP cases were non neoplastic lesions (oncocytic hyperplasia). Among the eight unspecified SUMP cases, four turned out to be malignant neoplasm, one benign neoplasm and two non-neoplastic after pathological examination. One of these two non-neoplastic lesions was an atrophic sialadenitis which is one of the pitfalls of FNA cytological examination since ICC is not contributive in these cases [6]. The other non-neoplastic lesion was a fibrous nodule. Numerous fibroblasts were present in this lesion and these cells have been confused with atypical cells on cytological smears. The RON and ROM for SUMP category, oncocytic SUMP subtype and unspecified SUMP subtype are consistent with the MSRSGC and previous studies [3,6,14,15]. The SM category was also seldom used since it represented only 4.9% of our cases. Among the 16 SM cases, five cases were not malignant after histological analysis. Two of the cases suspicious for lymphoma turned out to be non-neoplastic (one reactive lymph node and one lymphoepithelial cyst). The three other cases which were suspicious for epithelial malignancy were finally diagnosed as non-neoplastic (myofibroblastic granuloma) or benign neoplasm (two PA). Though, the ROM for SM is consistent with the MSRSGC (68.8% vs. 60%) and with the literature (68,8% vs. 79%, 59%, 58%) [9,11,16].

As most of the samples were provided as smears, we systematically stained the slides which contained the most cells with May-Grünwald-Giemsa (MGG). This allows good differentiation between the cells and the matrix, which is particularly useful for visualising matrix-rich tumours such as PA, adenoid cystic carcinomas and basal cell adenomas. Both MGG and Papanicolaou stain the mucus present in mucoepidermoid carcinomas. We observed that oncocytic cells present in Warthin’s tumours displayed characteristic orange-stained cytoplasms with Papanicolaou. When the lesion was evident, we stained the remaining slides either with MGG or with others stains. When the nature of the tumour was not evident, ICC or FISH analyses were performed on the remaining slides, as suggested by Jo et al., who reported a useful algorithm for the use of ancillary tests [17]. ICC was informative in 72.4% of cases. It was used to eliminate a metastasis in 11.8% of cases, discarding lung adenocarcinoma or thyroid carcinoma (TTF1-), epidermoid carcinoma (p63-), and melanoma (pS100, MelanA, HMB45). However, for some tumours such as squamous cell carcinoma, adenocarcinoma, and neuroendocrine small cell carcinoma, it is sometimes difficult to determine whether the tumour originated from the salivary gland or whether it is an intra-glandular lymph node metastasis. The clinical data is of special interest in these cases. ICC helped to differentiate BN/LG-M lesion from HG-M neoplasms in 36.8% of cases and provided the definitive diagnosis in 23.7% of cases (either by negative pancytokeratin staining indicating the absence of epithelial cells, the presence of mixed B and T reactive lymphocytes or the low percentage (<5%) of Ki67 positive cells). It was not informative in 27.6% of cases predominantly due to the presence of insufficient cells on the remaining slides available for analysis.

Our study used a limited panel of antibodies which could be extended to include EpCAM and LEF-1 for example. EpCAM expression has been shown in 97.6% of adenoid cystic carcinomas and its intensity is correlated with a poor prognosis but it cannot be used for a differential diagnosis on cytological samples [18]. LEF-1 has been shown to be positive in benign basaloid neoplasm tests [19]. Griffith et al., have developed a user-friendly pattern approach for the sequential use of ancillary tests [20]. According to the staining patterns obtained, a combination of immunostains useful for any subsequent characterisation is proposed. However, the limitation of the Griffith et al., approach is the limiting quantity of remaining sample which is available for analysis. ICC is more robust when performed on cellblocks, which necessitate sufficient amounts of material.

We observed that 13/37 salivary gland lesions identified as malignant were indeed metastases. It is generally difficult to differentiate between a primary and a secondary lesion. Indeed, in a recent review, Pastore et al., reported that 71% of salivary gland metastases in their institution arose from cutaneous head and neck tumours, 12% from the upper digestive tract, 12% from locations out of cervical region and 5% remained from unknown origin [21]. Wang et al., showed that metastatic squamous cell carcinoma from all sites (47.3%) and melanoma (36.4%) constituted the majority of secondary malignancies of salivary glands [22]. This is very similar to our observations, as 13 metastatic lesions in our study were identified as metastasis on cytology while 4/23 HG-M turned out to be metastasis on the pathological examination. Overall, 7/17 of the metastatic lesions originated from head and neck tumours, 5/17 from lung tumours (4 adenocarcinomas, 1 small cell carcinomas), 1/17 from urothelial carcinoma, 1/17 from Merckel cell carcinoma, 1/17 from melanoma and for 2/17 we were unable to identify the origin of the primary tumour. The presence of lymphocytes admixed with cancer cells is usually suggestive of a metastasis. However, the clinical history is the most informative indicator as it guides the choice of the most appropriate ICC tests to perform.

FISH analysis provided a definitive diagnosis in 33.3% of cases. It is very useful to definitively identify some tumours such as adenoid cystic carcinomas, secretory carcinomas, mucoepidermoid carcinomas and PA when they display unusual features. As the matrix is auto-fluorescent, FISH can be difficult to interpret when the matrix is very abundant. In such cases, it can only be interpreted when the actual diagnostic sample is positive. As we have already shown, the very high specificity of FISH allowed us to establish definitive diagnoses specifically in cases of adenoid cystic carcinomas, secretory carcinomas and mucoepidermoid carcinomas [5]. It is less useful for PA due to their easier cytological identification and because *PLAG1* gene rearrangement is present in only 60% of cases and because it does not differentiate benign from malignant forms. Using ICC and/or FISH analysis, the ROM for the M category was determined to be 100%. Park et al., observed a similar risk for this category, in accordance with the MSRSGC [4].

## 4. Materials and Methods

### 4.1. Cytological Samples

From February 2014 to March 2018, 328 salivary gland FNAs from 314 patients were received by our laboratory. The FNAs were performed using a 25-gauge needle. The clinician collected most of the samples when the patient consulted for an enlargement of a salivary gland. In some cases, a radiologist performed the puncture under guided ultrasound. Samples were received by the laboratory predominantly as air-dried smears, and/or sometimes as liquid based cytology (Hologic). For smears, one slide was systematically stained with May-Grünwald-Giemsa (MGG) in order to establish a diagnosis. If the diagnosis could be established, the remaining slides were stained with MGG or if necessary with Papanicolaou, Periodic Acid-Schiff (PAS) or Alcian blue. If the diagnosis was not evident, the remaining slides were used for ICC and/or FISH.

### 4.2. Cytological Classification

The cytological diagnoses were reviewed and retrospectively classified using the MSRSGC. They were classified as follows: non-diagnostic (ND), non-neoplastic (NN), atypia of undetermined significance (AUS), benign neoplasm (BN), salivary gland neoplasm of uncertain malignant potential (SUMP), suspicious for malignancy (SM) and malignant (M). For each diagnosis, we reported whether ICC and/or FISH was performed. The risk of neoplasm (RON) and the risk of malignancy (ROM) were calculated according to the definitive pathological diagnosis for all cytological categories.

### 4.3. Immunocytochemistry

Immunocytochemistry (ICC) analyses using different antibodies (listed in Table 5) were performed on the Dako Autostainer system or the Ventana Benchmark automated staining system according to the manufacturers’ recommendations. A polymer linked to a peroxidase system with DAB was used to amplify and visualize the signal. ICC results were classified as being either not contributing for the diagnosis, eliminating metastases of secondary tumours, differentiating Benign/LG-M from HG-M or providing the definitive diagnosis.

### 4.4. FISH

The FISH probes used in our laboratory as well as the FISH procedure have been described previously [5]. The result of FISH was classified as either not contributing to the diagnosis (because of a negative result), not helpful (negative) or providing the definitive diagnosis.

### 4.5. Histopathological Results Classification

When available, histopathological results were correlated with cytology for all of the categories. Histopathological results were separated into non-neoplastic, benign neoplasm or malignant.

## 5. Conclusions

In conclusion, the MSRSGC is more effective when specific features of neoplasms can be identified. Ancillary studies help to further characterise salivary gland tumours and thereby increase the accuracy of MSRSGC.

## Figures and Tables

**Table 1:** Classification of cytological results and correlation with histological results. *RON: risk of neoplasm; ROM: risk of malignancy.*

Cytopathology				Histopathology				RON		ROM	
Milan System Category		Number of Cases (%)	Number of Cases per Category (%)	Not Performed	Non-Neoplastic	Benign Neoplasm	Malignant	RON (%)	RON per Category (%)	ROM (%)	ROM per Category (%)
Non-Diagnostic (ND)	Pauci-cellular	55 (16.8)	84 (25.6)	24	2	20	9	29/31 (93.5)	38/47 (80.9)	9/31 (29)	16/47 (34)
	Cystic content	25 (7.6)		13	3	6	3	9/12 (75)		3/12 (25)	
	Lymphocytes	4 (1.2)		-	-	-	4	4/4 (100)		4/4 (100)	
Non-Neoplastic (NN)	Normal lymph node	7 (2.1)	27 (8.2)	5	2	-	-	0/9 (0)	1/9 (11.1)	0/9 (0)	0/9 (0)
	Reactive lymph node/Inflammation	12 (3.7)		10	2	-	-	0/9 (0)		0/9 (0)	
	Lithiasis/sialadenitis	1 (0.3)		1	-	-	-	0/9 (0)		0/9 (0)	
	Cyst	7 (2.1)		2	4	1	-	1/9 (11.1)		0/9 (0)	
Atypia of Undetermined Significance (AUS)		4 (1.2)	4 (1.2)	2	-	2	-	2/2 (100)	2/2 (100)	0/2 (0)	0/2 (0)
Benign Neoplasm (BN)	Pleomorphic adenoma	89 (27.1)	145 (44.2)	18	-	67	4	71/71 (100)	105/105 (100)	4/71 (5.63)	4/105 (3.09)
	Warthin’s tumour	55 (16.8)		21	-	34	-	34/34 (100)		0/34 (0)	
	Schwanomma	1 (0.3)		1	-	-	-	-		-	
Salivary Gland Neoplasm of Uncertain Malignant Potential (SUMP)	Oncocytic	7 (2.1)	15 (4.6)	3	2	1	1	2/4 (50)	7/11 (63.6)	1/4 (25)	5/11 (45.5)
	Unspecified	8 (2.4)		1	2	1	4	5/7 (71.4)		4/7 (57.1)	
Suspicious for malignancy (SM)	Epithelial	6 (1.8)	16 (4.9)		1	2	3	5/6 (83.3)	13/16 (81.3)	3/6 (50)	11/16 (68.8)
	Lymphoma	10 (3.1)		-	2	-	8	8/10 (80)		8/10 (80)	
Malignant (M)	Low-grade	1 (0.3)	37 (11.3)	-	-	-	1	1/1 (100)	26/26 (100)	1/1 (100)	26/26 (100)
	High-grade	23 (7)		4	-	-	19	19/19 (100)		19/19 (100)	
	Metastasis	13 (4)		7	-	-	6	6/6 (100)		6/6 (100)	
Total		328 (100)	328 (100)	112 (34.2)	18 (5.5)	136 (41.5)	62 (18.9)	-			

**Table 2:** Contributions of immunocytochemistry and FISH to the definitive diagnosis. ICC: Immunocytochemistry; LN: lymph node; LG-M: Low-grade malignant; HG-M: High-grade malignant.

Cytopathology			Immunocytochemistry					FISH			
Milan System Category		Total Number of Cases	Total Number of Cases with ICC (%)	Did not Contribute to Diagnosis	Eliminated Metastasis of Secondary Tumours	Differentiated Benign/LG-M from HG-M	Provided the Diagnosis	Total Number of Cases with FISH (%)	Did not Contribute to Diagnosis	No Help (Negative)	Provided the Diagnosis
Non-Diagnostic (ND)	Pauci-cellular	55	**2/55 (3.6%)**	1 (50%)		1 (50%)					
	Cyst content	25	**3/25 (12%)**	1 (33.3%)	1 (33.33%)	1 (33.3%)		**1/25 (4%)**		1 (100%)	
	Lymphocytes	4	**3/4 (75%)**	2 (66.7%)			1 (33.3%)				
Non-Neoplastic (NN)	Normal LN	7	**3/7 (42,9%)**		3 (100%)						
	Inflammation	12	**6/12 (50%)**		1 (16,7%)	5 (83,3%)					
	Sialadenitis	1	**1/1 (100%)**			1 (100%)					
	Cyst	7	**1/7 (14.3%)**				1 (100%)				
Atypia of undetermined significance (AUS)		4	**3/4 (75%)**	3 (100%)				**1/4 (25%)**	1 (100%)		
Benign neoplasm (BN)	Pleomorphic adenoma	89	**12/89 (13.5%)**	2 (16.7%)		9 (75%)	1 (8.3%)	**32/89 (36%)**	7 (21.9%)	14 (43.8%)	11 (34.4%)
	Warthin’s tumour	55	**1/55 (1.8%)**			1 (100%)		**1/55 (1.82%)**		1 (100%)	
	Other	1									
Salivary Gland Neoplasm of Uncertain Malignant Potential (SUMP)	Oncocytic	7	**3/7 (42.9%)**	2 (66.7%)		1 (33.3%)		**3/7 (42.9%)**	1 (33.3%)	2 (66.7%)	
	Undetermined	8	**2/8 (25%)**	1 (50%)		1 (50%)		**3/8 (37.5%)**	1 (33.3%)	1 (33.3%)	1 (33.3%)
Suspicious for malignancy (SM)	Epithelial	6	**3/6 (50%)**	3 (100%)				**3/6 (50%)**		2 (66.7%)	1 (33.3%)
	Lymphoma	10	**7/10 (70%)**	2 (28.6%)	2 (28.6%)	1 (14.3%)	2 (28.6%)				
Malignant (M)	Low-grade	1	**1/1 (100%)**				1 (100%)	**1/1 (100%)**			1 (100%)
	High-grade	23	**17/23 (73.9%)**	3 (17.6%)	2 (11.8%)	6 (35.3%)	6 (35.3%)	**2/23 (8.7%)**			2 (100%)
	Metastasis	13	**8/13 (61.5%)**	2 (25%)		1 (12.5%)	5 (62.5%)				
Total		**328**	**76/328 (23,2%)**	**21/76 (27.6%)**	**9/76 (11.8%)**	**28/76 (36.8%)**	**18/76 (23.7%)**	**48/328 (14.6%)**	**10/48 (20.8%)**	**22/48 (45.8%)**	**16/48 (33.3%)**

**Table 3:** Details of the nine cases in which ICC allowed to eliminate a metastasis. ND: Non-Diagnostic; NN: Non-Neoplastic; SM: Suspicious for Malignancy; M: Malignant; HG: High-grade; neg: negative; pos: positive; DLBCL: Diffuse large B-cell lymphoma; GC: germinal center.

Patient	Medical History of Neoplasm	Milan System Category	Cytological Diagnosis	Immunochemistry			Histological Diagnosis
				Antibody Used	Results	Justification	
58	None	ND	Cyst content	TTF1	neg	eliminated metastasis of potential papillary thyroid carcinoma	Mucoepidermoid carcinoma ex PA
85	Tongue squamous cell carcinoma diagnosed 1 month before	NN	reactive lymph node	AE1/AE3	neg	eliminated metastasis of the squamous cell carcinoma previously diagnosed	not performed
				CD3	pos		
				CD20	pos		
86	Metastatic lung adenocarcinoma diagnosed 3 month before	NN	normal lymph node	AE1/AE3	neg	eliminated metastasis of the lung adenocarcinoma previously diagnosed	not performed
				CD3	pos		
				CD20	pos		
				CK7	neg		
				TTF1	neg		
93	None	NN	normal lymph node	CD3	pos	eliminated metastasis of a carcinoma	not performed
				CD20	pos		
				CK7	neg		
94	Papillary thyroid carcinoma diagnosed in 1996	NN	normal lymph node	TTF1	neg	eliminated metastasis of the papillary thyroid carcinoma previously diagnosed	Normal lymph node
284	None	SM	lymphoma	AE1/AE3	neg	eliminated metastasis of a carcinoma	Follicular lymphoma grade 1–2
285	None	SM	lymphoma	AE1/AE3	neg	eliminated metastasis of a carcinoma	DLBCL non-GC
297	None	M (HG)	undifferenciated carcinoma	TTF1	neg	eliminated metastasis of a lung adenocarcinoma	salivary duct carcinoma
				p63	pos		
298	None	M (HG)	undifferenciated carcinoma	TTF1	neg	eliminated metastasis of a lung adenocarcinoma	salivary duct carcinoma
				p63	pos		

**Table 4:** Cases with Immunocytochemistry and FISH. BN: Benign-Neoplasm; SUMP: Salivary Gland Neoplasm of Uncertain Malignant Potential; M: Malignant; LG: Low-grade HG: High-grade; PA: pleomorphic adenoma; AdCC: adenoid cystic carcinoma; SC: secretory carcinoma; ADK, NOS: adenocarcinoma not otherwise specified; neg: negative; pos: positive.

Patient	Milan System Category	Cytological Diagnosis	Histological Diagnosis	Immunocytochemistry			FISH		
				Antibody Used	Results	Contribution	Probe Used	Results	Contribution
116	BN	PA	PA	Ki67	1% pos	Eliminated HG-M	PLAG1	neg	No help
117	BN	PA	PA	Ki67	2% pos	Eliminated HG-M	PLAG1	neg	No help
118	BN	PA	not performed	p63	pos	provided the diagnosis	PLAG1	pos	Provided the diagnosis
119	BN	PA	not performed	Ki67	2% pos	Eliminated HG-M	PLAG1	pos	Provided the diagnosis
120	BN	PA	not performed	Ki67	1% pos	Eliminated HG-M	PLAG1	pos	Provided the diagnosis
121	BN	PA	PA	Ki67	3% pos	Eliminated HG-M	PLAG1	pos	Provided the diagnosis
264	SUMP	SUMP with Oncocytic features	Cellular PA with oncocytyc cells	Ki67	3% pos	Eliminated HG-M	PLAG1	neg	No help
293	M (HG)	AdCC	AdCC	Ki67	10% pos	In favor of HG-M	MYB	pos	Provided the diagnosis
							PLAG1	neg	
294	M (HG)	AdCC	AdCC	p63	pos	did not contribute to diagnosis	MYB	pos	Provided the diagnosis
295	M (LG)	SC	SC	MAM	30% pos	provided the diagnosis	ETV6	pos	Provided the diagnosis
299	M (HG)	ADK, NOS	metastasis of lung adeno-carcinoma	Ki67	20% pos	In favor of HG-M	ETV6	neg	No help

**Table 5:** Antibodies and corresponding protocols used (antigen retrieval, concentration).

	Provider	Antigen Retrieval	Automate	Dilution
CD3	Roche	pH9	Ventana, Roche	RTU
CD79a	Roche	pH9	Ventana, Roche	RTU
CD15	Roche	pH9	Ventana, Roche	RTU
CD30	Roche	pH9	Ventana, Roche	RTU
CD45	Roche	pH9	Ventana, Roche	RTU
CD68-PGM1	Clinisciences	No	Ventana, Roche	1/100
CD56	Roche	pH9	Ventana, Roche	RTU
AE1/AE3	Agilent	No	Autostainer, Dako	RTU
p63	Agilent	pH9	Autostainer, Dako	1/50
p40	Dako	pH6	Autostainer, Dako	1/200
CK5/6	Agilent	No	Autostainer, Dako	1/100
CK7	Agilent	No	Autostainer, Dako	1/400
CK20	Agilent	No	Autostainer, Dako	1/200
TTF1	Agilent	pH6	Autostainer, Dako	RTU
Chromogranin	Agilent	No	Autostainer, Dako	1/100
Synpatophysin	MM France	pH9	Autostainer, Dako	RTU
Ki67	Agilent	pH6	Autostainer, Dako	1/50
Mammaglobin	Dako	No	Autostainer, Dako	RTU
Desmin	Agilent	No	Autostainer, Dako	1/100
Vimentin	Dako	No	Autostainer, Dako	RTU
Smooth Muscle Actin	Agilent	No	Autostainer, Dako	RTU
Androgen Receptor	Dako	pH9	Autostainer, Dako	1/200
MelanA	Agilent	No	Autostainer, Dako	RTU
	(1)			

RTU: ready-to-use.
